# Correction: Prostate-specific membrane antigen cleavage of vitamin B9 stimulates oncogenic signaling through metabotropic glutamate receptors

**DOI:** 10.1084/jem.2017105211212017c

**Published:** 2018-01-02

**Authors:** Charalambos Kaittanis, Chrysafis Andreou, Haley Hieronymus, Ninghui Mao, Catherine A. Foss, Matthias Eiber, Gregor Weirich, Palak Panchal, Anuradha Gopalan, Juan Zurita, Samuel Achilefu, Gabriela Chiosis, Vladimir Ponomarev, Markus Schwaiger, Brett S. Carver, Martin G. Pomper, Jan Grimm

Vol. 215, No. 1, January 2018. https://doi.org/10.1084/jem.20171052

JEM regrets that in the original version of this paper, the labels appeared incorrectly in Fig. 7 A and Fig. S5 G as the result of a production error.

All versions of this article have been corrected.

